# PAUNet: A Lightweight Network for Real-Time Underwater Image Enhancement

**DOI:** 10.3390/s26206546

**Published:** 2026-10-15

**Authors:** Kelong Yin, Dongjin Jiang, Hongyang Xu, Yongqing Zeng, Mingyu Qiu, Xiaoping Wang

**Affiliations:** 1 Ocean College, Zhejiang University, Zhoushan 316000, China; 3190101418@zju.edu.cn (K.Y.);; 2 Donghai Laboratory, Zhoushan 316000, China

**Keywords:** underwater image enhancement, lightweight network, partial convolution, axial depthwise convolution, spatial attention

## Abstract

Underwater images are degraded by wavelength-dependent absorption and scattering, while enhancement on resource-constrained platforms requires a small model and low inference latency. We propose PAUNet, a lightweight encoder–decoder network combining partial axial depthwise convolution (PADW), multiscale axial spatial adaptive attention (MASA), and attention-guided skip connections. PADW filters half of the feature channels and preserves the remainder through an identity branch. MASA combines three axial scales using spatial branch attention. A joint objective constrains reconstruction, structure, perceptual features, and color. PAUNet achieves PSNR values of 21.508, 28.696, and 28.632 dB on UIEB, EUVP, and LSUI, respectively, with SSIM values of 0.751, 0.843, and 0.895. Among the compared methods, PAUNet has the highest PSNR on EUVP and LSUI, ranks second in UIEB PSNR, and achieves the highest LSUI SSIM. PAUNet contains 64,495 parameters, requires 0.493 GFLOPs, and runs at 288.95 frames per second on an NVIDIA RTX 3090. On an NVIDIA Jetson Xavier NX, TensorRT FP16 inference reaches 74.55 frames per second at 256 × 256 resolution and batch size one. The comparisons and ablations evaluate this compact architecture for underwater image enhancement.

## 1. Introduction

Underwater optical images commonly exhibit color casts, low contrast, and blurred details because light is absorbed and scattered during propagation [1]. Wavelength-dependent attenuation alters the relative strengths of the color channels, while backscatter reduces visibility. These effects limit the usefulness of visual measurements for underwater localization, robotic perception, and environmental monitoring [2,3]. Image enhancement is, therefore, an important component of underwater visual sensing. For platforms with limited computing and energy resources, enhancement quality must be considered together with model size and inference latency.

Existing solutions include physical restoration, statistical enhancement, and learned image-to-image mappings. Physical methods estimate scene radiance using assumptions about transmission and background illumination, whereas statistical methods adjust color and contrast directly. Learning-based models can recover complex degradations from data; however, their computational requirements vary considerably. A compact model must preserve spatial detail and correct color without relying on a large feature hierarchy or expensive global operations.

We propose PAUNet, a partial axial underwater image enhancement network. Its encoder–decoder architecture allocates spatial filtering to a subset of channels, combines multiple receptive-field scales at the bottleneck, and recalibrates skip features before reconstruction. The design targets an efficient representation of underwater degradation while retaining the detailed information needed by the decoder. The main contributions are as follows:

- A partial axial depthwise convolution (PADW) module combines parallel horizontal and vertical depthwise filters with a channel bypass. Filtering half of the channels halves the axial spatial-convolution cost relative to the full-channel operator;
- A multiscale axial spatial adaptive attention bottleneck (MASA) selects spatial responses from three axial scales. Attention-guided skip connections complement the bottleneck by refining shallow features;
- A compact enhancement network combines these components with pixel, structure, perceptual, and color losses. Comparisons on three datasets and targeted ablations evaluate its reconstruction quality, parameter count, and measured inference speed.

## 2. Related Work

### 2.1. Physical and Statistical Enhancement

Physical restoration methods use image-formation models to estimate the degradation process. Dark channel prior [4] motivated underwater adaptations that estimate depth or transmission from single images [5]. Maximum attenuation identification provides another route to transmission estimation and color restoration [6]. Polarization-based approaches use additional optical measurements to separate useful scene information from scattering, including light-field/polarization imaging [7] and periodic integration-based polarization differential imaging [8]. Their performance depends on the imaging conditions, available measurements, and accuracy of the estimated parameters.

Statistical enhancement operates without explicitly inverting an underwater image-formation model. Iqbal et al. adjusted color and intensity through an integrated color model [9]. Fu et al. introduced a Retinex-based strategy for single underwater images [10], and Ancuti et al. fused complementary enhanced inputs at multiple scales [11]. Such methods offer practical ways to improve visibility; however, fixed assumptions about image statistics can produce residual color casts or excessive local contrast when scene conditions change.

### 2.2. Learning-Based Underwater Image Enhancement

Learning-based approaches estimate the mapping from degraded images to enhanced outputs using paired or unpaired data. Water-Net combines preprocessed inputs with learned confidence maps and was introduced with the UIEB benchmark [12]. FUnIE-GAN uses adversarial and perceptual supervision for fast enhancement [13]. Ucolor combines multiple color–space representations with medium transmission guidance [14]. The U-shape Transformer introduces channel and spatial modeling for underwater enhancement and provides the LSUI dataset [15]. These studies show the value of complementary feature representations; however, the resulting quality–efficiency trade-off depends on the backbone and its operators.

### 2.3. Lightweight Network Design

Efficient convolutional networks reduce spatial and channel-mixing costs in different ways. MobileNet separates depthwise spatial filtering from pointwise channel mixing [16], while ShuffleNet combines grouped pointwise convolution with channel shuffling [17]. For underwater enhancement, LU2Net integrates axial depthwise convolution and channel attention into a compact U-shaped network [18]. FasterNet further demonstrates the potential of computing spatial features on only part of the channel dimension [19]. PAUNet extends this line of compact axial encoder–decoder designs by combining partial-channel axial filtering, a multiscale bottleneck, and spatial recalibration of skip features. Its contribution is evaluated through these specific architectural changes and their measured quality–cost trade-offs.

## 3. Materials and Methods

### 3.1. Network Architecture

**Figure 1:**
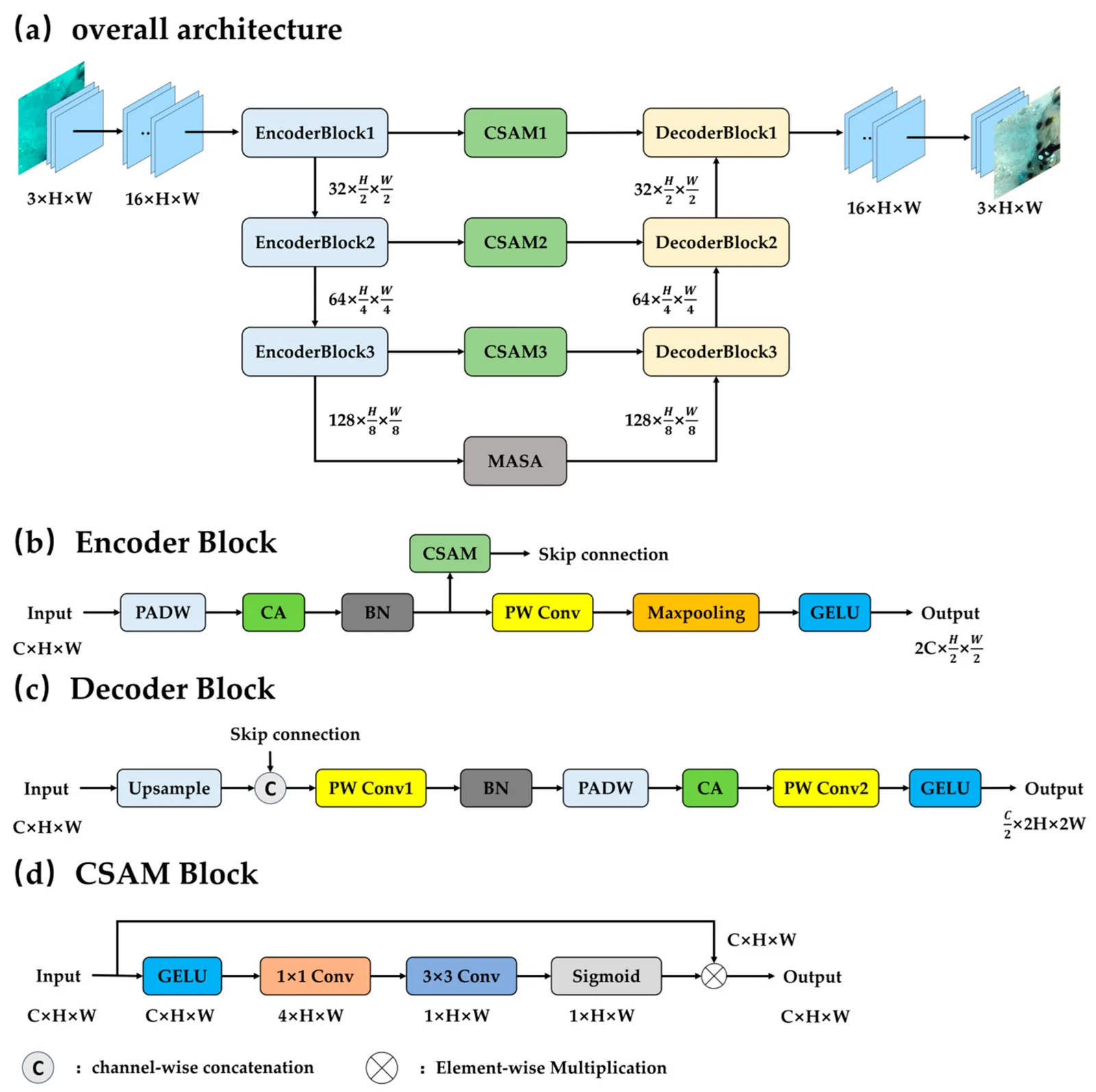
PAUNet architecture: (**a**) encoder–decoder network; (**b**) encoder block; (**c**) decoder block; and (**d**) channel–spatial attention module (CSAM).

PAUNet contains a shallow feature extractor, three encoder blocks, a MASA bottleneck, three decoder blocks, and an RGB reconstruction layer (Figure 1). A 5 × 5 convolution with GELU activation maps the input image I with dimensions 3 × H × W to 16 feature channels:

F_0_ = GELU(Conv_5×5_(I)). (1)

Each encoder block first applies PADW, channel attention (CA), and batch normalization (BN). The resulting feature S_l_ is retained for a skip connection before any channel expansion or downsampling. A pointwise convolution then doubles the channel dimension, followed by stride-2 max pooling and GELU activation:

S_l_ = BN(CA(PADW(F_l_))), (2)

F_l+1_ = GELU(MaxPool(Conv_1×1_(S_l_))). (3)

In PAUNet, each CA module averages the spatial dimensions, projects C channels to C/2 and back to C through two bias-free 1 × 1 convolutions, and applies ReLU, followed by sigmoid gating. The three scales, therefore, use 16 → 8 → 16, 32 → 16 → 32 and 64 → 32 → 64 channels in both the encoder and decoder. The channel weights multiply the input features.

The three post-downsampling encoder outputs have 32, 64, and 128 channels at resolutions H/2 × W/2, H/4 × W/4, and H/8 × W/8, respectively. The corresponding skip features are extracted before pointwise expansion and pooling, with dimensions of 16 × H × W, 32 × H/2 × W/2, and 64 × H/4 × W/4. MASA processes the deepest feature at the lowest resolution. In the decoder, bilinear interpolation doubles the spatial resolution without adding trainable parameters. Each upsampled feature is concatenated with the recalibrated skip feature at the same resolution, reduced by pointwise convolution, and refined by BN, PADW, and CA. A second pointwise convolution and GELU produce the decoder output:

U_l_ = Concat(Up(D_l+1_), CSAM(S_l_)), (4)

D_l_ = GELU(PW_2_(CA(PADW(BN(PW_1_(U_l_)))))). (5)

Here, PW denotes a 1 × 1 convolution and Concat denotes concatenation along the channels. A final 1 × 1 convolution maps the last decoder feature to the three-channel enhanced image Î.

For a skip tensor S with dimensions C × H × W, CSAM first forms G = GELU(S), then P = Conv1 × 1(G) with dimensions 4 × H × W, and A = σ(Conv3 × 3(P)) with dimensions 1 × H × W (Figure 1d). The 3 × 3 projection uses one-pixel padding. The attention map multiplies the original skip tensor:

CSAM(S) = S ⊙ σ(Conv_3×3_(Conv_1×1_(GELU(S)))). (6)

In Equation (6), the indexed rule is Y[c,h,w] = A [1,h,w]S[c,h,w] for every c = 1,…,C. The same spatial coefficient is broadcast across channels, preserving C × H × W. CSAM provides spatial recalibration, while CA adjusts channel responses. The skip features retain information from before the next downsampling operation.

### 3.2. Partial Axial Depthwise Convolution

**Figure 2:**
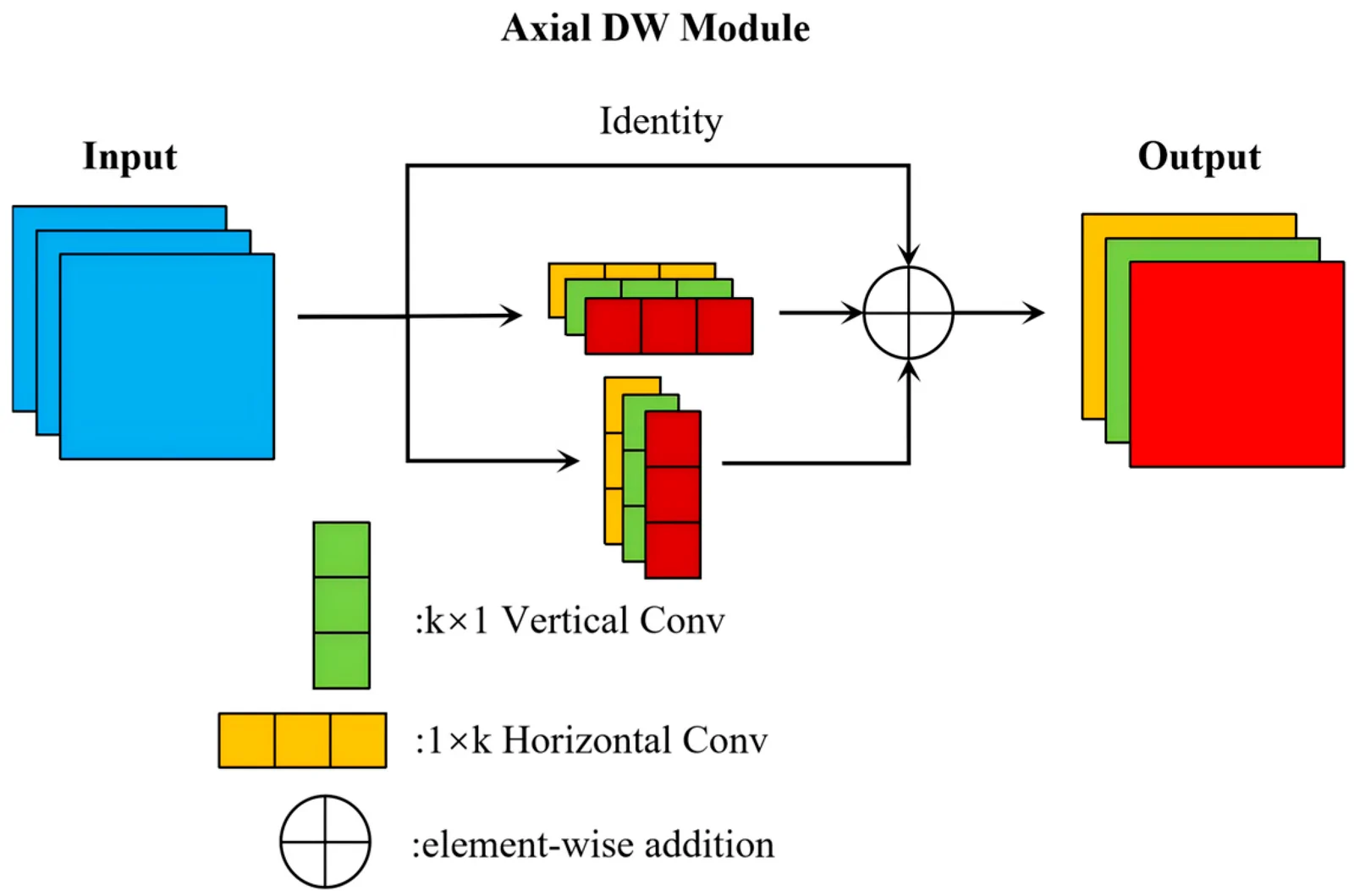
Full-channel axial depthwise convolution with parallel vertical k × 1 and horizontal 1 × k filters.

Axial depthwise convolution provides the spatial-filtering operation used in PADW. As illustrated in Figure 2, a k × 1 depthwise filter operates along the vertical direction and a 1 × k depthwise filter operates along the horizontal direction. Both filters receive the same input and operate independently within each channel. Their responses are added to the identity branch shown in Figure 2, following the residual axial filtering used in LU2Net [18]. This operator aggregates information along the two orthogonal directions without applying a dense k × k filter.

**Figure 3:**
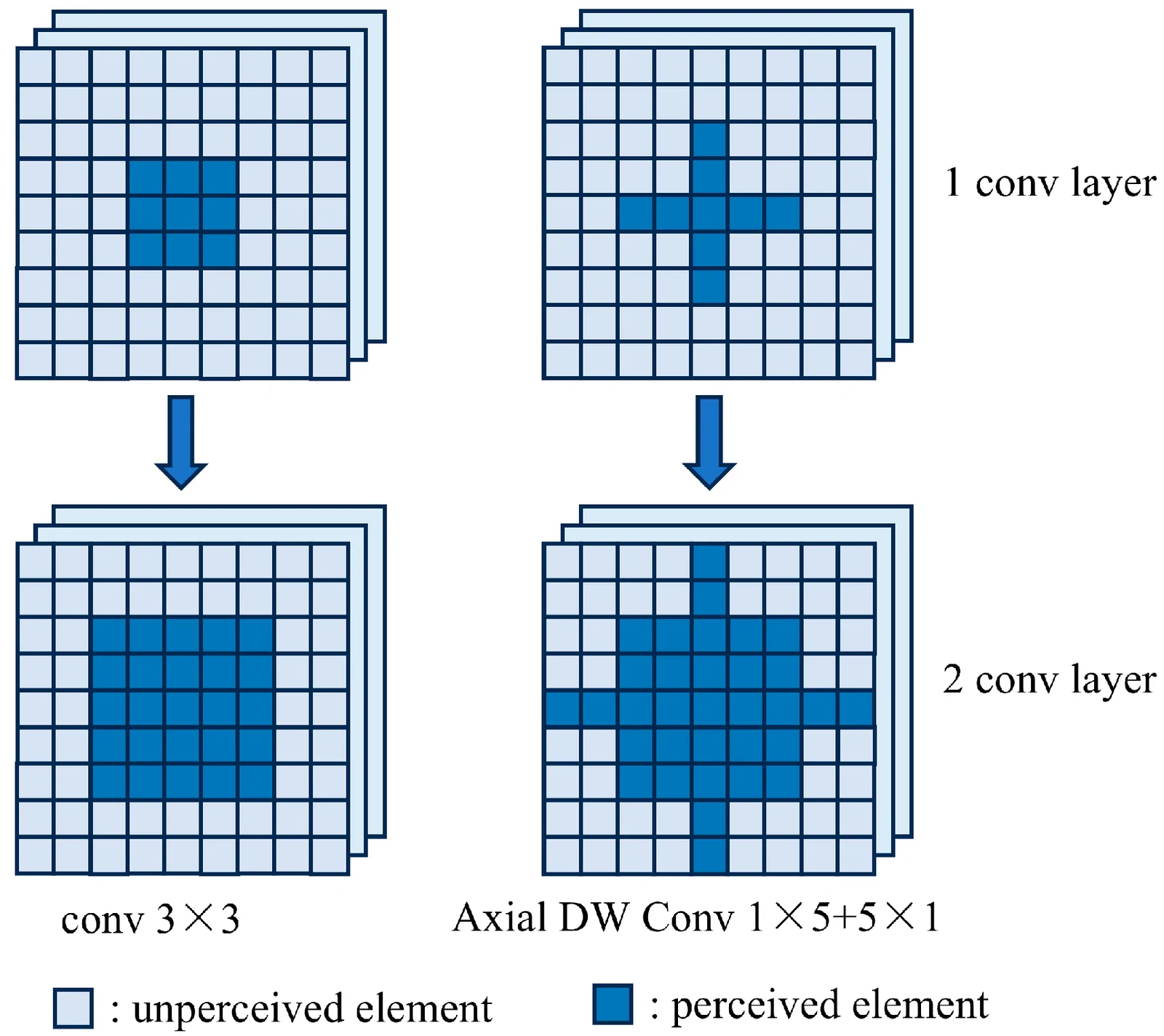
Spatial support of stacked standard 3 × 3 convolutions and parallel 5 × 1/1 × 5 axial filters. Shaded positions indicate theoretical spatial support.

Figure 3 compares stacked standard 3 × 3 convolutions with axial filters with a size of 5 × 1 and 1 × 5. A single axial layer samples a cross-shaped set of positions within a 5 × 5 bounding region, whereas a standard 3 × 3 convolution samples all nine positions in its smaller region. Stacking axial layers enables information to propagate through successive horizontal and vertical paths. The comparison illustrates the support of these particular kernel choices; axial filtering does not enlarge the bounding region relative to a dense convolution with the same kernel length.

Along a path passing through L such spatial operators, the theoretical receptive-field extent in each direction is R = 1 + L(k − 1), assuming no intervening operation changes the sampling stride. For k = 5, one layer has a 5 × 5 bounding region and two layers extend this region to 9 × 9. The positions inside the bounding region are not necessarily all covered: two parallel axial layers still have a different support pattern from two dense 5 × 5 layers. This description concerns the theoretical support of the convolution path; the partial-channel bypass and network downsampling must also be considered when interpreting the complete network.

**Figure 4:**
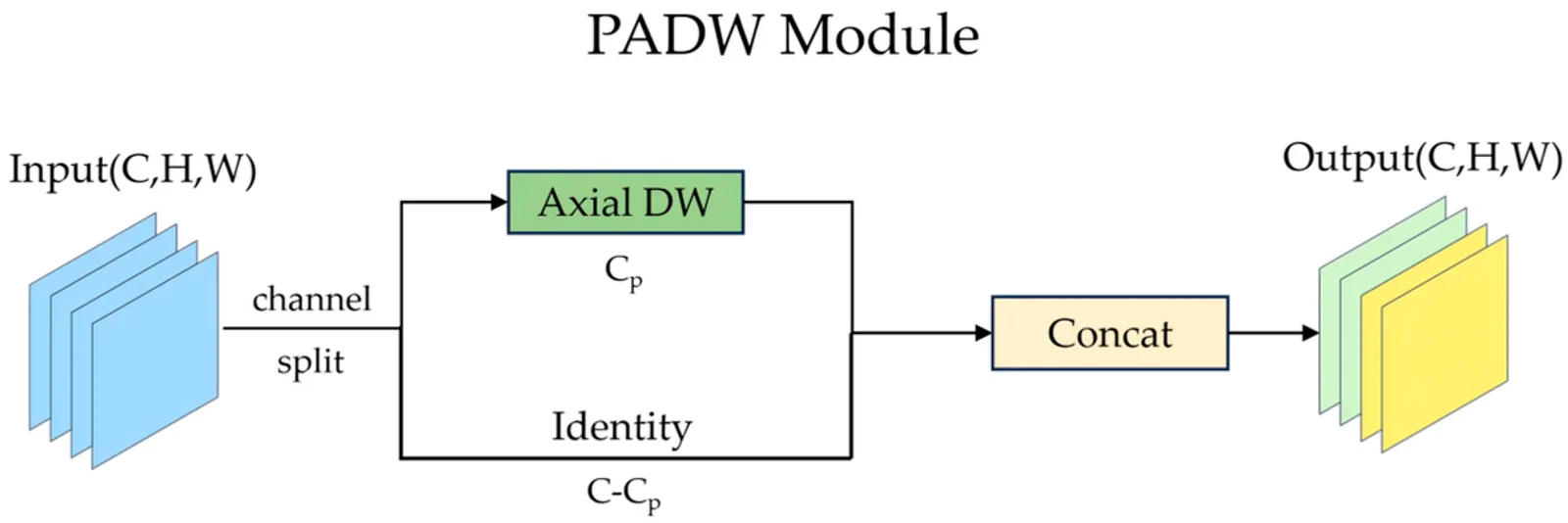
PADW module: channel splitting, residual parallel axial filtering in the processed branch, an identity bypass, and channel concatenation.

PADW applies axial filtering to a subset of channels (Figure 4). For X with dimensions C × H × W, let C_p_ = floor(rC), where r is the requested partial ratio. Splitting along channels yields X_p_ with C_p_ channels and Xᵦ with C−C_p_ channels. The processed branch adds parallel axial responses to its input, while the bypass is unchanged:

X′_p_ = X_p_ + DW_k×1_(X_p_) + DW_1×k_(X_p_), (7)

PADW(X) = Concat(X′_p_, X_b_). (8)

The default configuration uses k = 5 and r = 1/2. The processed branch, therefore, performs spatial filtering on half of the channels, while the other half follows the identity branch. Concatenation restores the original channel count and preserves the input spatial resolution. Adjacent pointwise convolutions mix information across channels, enabling filtered and bypassed features to interact in the surrounding network. This separation of spatial filtering and channel mixing reduces the cost of the axial operator while retaining an unfiltered feature pathway.

For a stride-one convolution with C input channels, C output channels, and an H × W output feature map, the dominant multiply–accumulate (MAC) counts of dense and depthwise k × k convolutions are:

MAC_conv_ = HWk^2^C^2^, MAC_DW_ = HWk^2^C (9)

The full-channel axial operator uses two depthwise filters of length k, whereas PADW applies those filters to C_p_ channels. Their spatial-convolution MAC counts are:

MAC_axial_ = 2HWkC, MAC_PADW_ = 2HWkC_p_. (10)

The exact PADW-to-axial MAC ratio is C_p_/C = floor(rC)/C; it equals r when rC is an integer. At r = 1/2 with even C, the axial spatial-convolution MAC count falls by 50%. For C = 128, k = 5, and H = W = 32, the full and partial operators require approximately 1.31 and 0.66 million MACs. These counts exclude bias, elementwise operations, normalization, activation, and data movement. Whole-network costs are reported separately in Section 4.3.2.

### 3.3. Multiscale Axial Spatial Adaptive Attention

**Figure 5:**
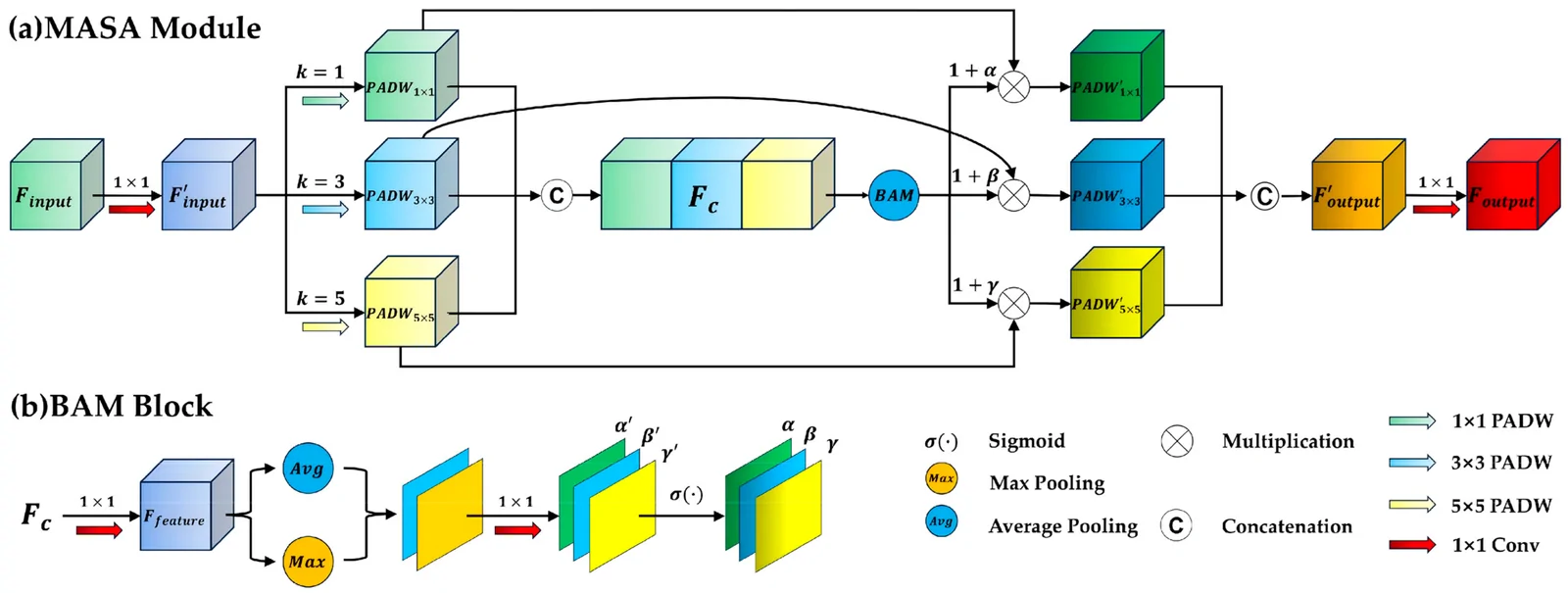
MASA bottleneck and branch attention: channel reduction, three axial scales, spatial attention generation, residual branch weighting, and output projection.

For PAUNet, MASA operates on the 128-channel bottleneck at H/8 × W/8 (Figure 5). A 1 × 1 projection reduces the channels from 128 to 24. Three PADW branches use kernel lengths k ∈ {1, 3, 5}; each filters 12 of its 24 channels and retains the other 12 through the identity branch:

Z = PW_in_(F), B_k_ = PADW_k_(Z). (11)

The three 24-channel branch outputs are concatenated into 72 channels. The attention path projects 72 to 64 channels by a 1 × 1 convolution. Channel-wise average and maximum pooling produce two spatial maps, which are concatenated and projected from two to three channels. A sigmoid generates one map per branch:

B = conv_1×1_(Concat(B_1_,B_3_,B_5_)), (12)

(A_1_, A_3_, A_5_) = σ(Conv(Concat(Avg_c_(B), Max_c_(B)))). (13)

Each attention map multiplies its corresponding branch through B′_k_ = B_k_ ⊙ (1 + A_k_). The weighted 24-channel outputs are concatenated into 72 channels and projected back to 128:

F_out_ = PW_out_(Concat(B′_1_, B′_3_, B′_5_)). (14)

The three attention maps retain the bottleneck spatial resolution and broadcast across their respective branch channels. Independent sigmoid gates scale each branch by a factor between one and two. The final pointwise projection fuses the three scales and restores the decoder input width.

### 3.4. Training Objective

The training objective combines Charbonnier reconstruction loss, CIELab and CIELCH color losses, VGG feature loss, and SSIM loss. Let Î_i_ and I_i_* denote the enhanced and reference images for sample i in a batch of N images. The Charbonnier loss is averaged over the batch, RGB channels, and spatial positions:

(15) $$L_{\mathrm{Char}}=\frac{1}{3\mathrm{NHW}}\sum_{i=1}^{N}\sum_{c=1}^{3}\sum_{p=1}^{\mathrm{HW}}\sqrt{{\left({\hat{I}}_{i,c}(p)-I_{i,c}^{*}(p)\right)}^{2}+\varepsilon^{2}}$$

Here, c indexes the RGB channels, p indexes the HW spatial positions, and H and W are the image height and width. RGB intensities are scaled to [0, 1] and ε = 10^−3^. The color terms compare the enhanced and reference images after conversion from RGB into three-channel color representations:

(16) $$L_{\mathrm{Lab}}=\frac{1}{3\mathrm{NHW}}\sum_{i=1}^{N}\|\mathrm{Lab}({Î}_{i})-\mathrm{Lab}(I_{i}^{*}){\|}_{2}^{2},$$

(17) $$L_{\mathrm{LCH}}=\frac{1}{3\mathrm{NHW}}\sum_{i=1}^{N}\sum_{p=1}^{\mathrm{HW}}[{\left({\Delta L}_{\mathrm{ip}}\right)}^{2}+{\left({\Delta C}_{\mathrm{ip}}\right)}^{2}+m_{\mathrm{ip}}{\left({\Delta h}_{\mathrm{ip}}\right)}^{2}].$$

Lab and LCH encode the same colors in Cartesian and polar coordinates. With C = √(a^2^ + b^2^) and h = atan2(b, a), the Lab chromatic error is (Δa)^2^ + (Δb)^2^ = (ΔC)^2^ + 2CC*[1 − cos(Δh)];C* and h* denotes the corresponding reference-image value, and the angular penalty depends on chroma. LCH separates radial and angular errors. In Equation (17), i indexes images and p indexes pixels; Δ denotes enhanced minus reference, Δh = atan2(sin(h − h*), cos(h − h*)), and m = 1 only when both chroma values satisfy C^2^ > 10^−6^; otherwise, m = 0. Lab coordinates and chroma retain their physical units; the hue is in radians, with no additional component-wise normalization. Perceptual supervision compares fixed ImageNet-pretrained VGG19 features [20]:

(18) $$L_{\mathrm{VGG}}=\frac{1}{NC_{j}H_{j}W_{j}}\sum_{i=1}^{N}\|\phi_{j}({Î}_{i})-\phi_{j}(I_{i}^{*}){\|}_{1}.$$

PAUNet uses frozen VGG19 relu4_4 features (index 26), evaluation mode, RGB means (0.485, 0.456, 0.406), and standard deviations (0.229, 0.224, 0.225). Feature error is the L1 mean. The structural term is L_SSIM_ = 1 − SSIM(Î, I*), averaged over the batch [21]. The reference objective is:

L = L_Char_ + 10^−7^L_Lab_ + 10^−3^L_LCH_ + 10L_VGG_ + 0.1L_SSIM_. (19)

The coefficients in Equation (19) remain fixed throughout training and define the relative weighting of the five losses. Section 4.3.3 assesses each term by removing it individually while retaining the remaining coefficients.

### 3.5. Datasets and Experimental Settings

Experiments were conducted using LSUI, UIEB, and EUVP. The LSUI collection contains 4279 image pairs [15], divided into 2995 training, 856 validation, and 428 test pairs using seed 3407, following a 7:2:1 allocation. PAUNet and the learning-based comparison methods were trained on the same LSUI training split. For full-reference evaluation, 100 image pairs were sampled without replacement from the 890 reference-paired UIEB images [12] using seed 3408. For EUVP [13], multiple paired subsets formed a single pool, from which 100 image pairs were sampled without replacement using seed 3409. These fixed UIEB-100, EUVP-100, and LSUI test sets were shared by all compared methods, including DNnet and FGDPA. Module and partial-ratio ablations used the same UIEB-100 subset; loss ablations used the same LSUI test split. The separate, unpaired UIEB Challenging-60 set was evaluated in full by all methods. These seeds specify data partitioning and sampling, rather than model initialization.

PAUNet was implemented in PyTorch 1.8.1 and trained on a PC equipped with an NVIDIA RTX 3090 GPU. Images were resized to 256 × 256 pixels. Training used a batch size of 16 for 100 epochs, with AdamW, an initial learning rate of 10^−3^, and weight decay of 10^−4^. ReduceLROnPlateau halves the learning rate when the loss on the LSUI validation split does not improve for five consecutive epochs. The loss weights follow Section 3.4. All ablation variants were retrained after the specified change. The baselines used the authors’ code and method-specific settings, with selected adjustments: LU2Net used 100 epochs and batch size 16 (500 epochs and batch size 1 in the released notebook), and FGDPA used 100 epochs (1000 recommended).

### 3.6. Evaluation Metrics

For all methods, PSNR and SSIM were computed on 256 × 256 RGB images with intensities in [0, 1] and no additional border cropping. PSNR was 10 log_10_(1/MSE), with MSE averaged over RGB channels and pixels. SSIM used an 11 × 11 Gaussian window (σ = 1.5), averaged over RGB channels and valid window locations. Reported scores are arithmetic means of per-image values on each fixed test subset. UIQM [22] and UCIQE [23] provided complementary no-reference measurements. UIQM combines colorfulness, sharpness, and contrast, whereas UCIQE combines chroma variability, luminance contrast, and mean saturation. Higher values are preferred within a consistent implementation. No-reference scores were interpreted alongside visual comparisons rather than as direct measurements of reconstruction fidelity.

## 4. Results

### 4.1. Comparison on Paired Datasets

The comparison includes three non-learning baselines, MLLE [24], WWPF [25], and WFAC [26], and nine learning-based baselines: FUnIE-GAN, Water-Net, USUIR [27], U-shape Transformer, CCL-Net [28], TUDA [29], LU2Net [18], DNnet [30], and FGDPA [31]. Table 1 compares these 12 baselines with PAUNet using PSNR and SSIM on UIEB, EUVP, and the LSUI test split. The baseline names follow the source implementations; “U-shape” denotes the U-shape Transformer. All 13 methods are evaluated on the same fixed test images at 256 × 256 resolution.

Table 1 shows that DNnet achieves the highest UIEB PSNR and SSIM, at 22.454 dB and 0.891. PAUNet ranks second in UIEB PSNR, at 21.508 dB—0.946 dB below DNnet; its SSIM of 0.751 ranks eighth among the 13 methods. On EUVP, PAUNet achieves the highest PSNR and SSIM, at 28.696 dB and 0.843, exceeding the second-highest PSNR of FUnIE-GAN by 0.190 dB, and the second-highest SSIM of DNnet by 0.007. On LSUI, PAUNet has the highest PSNR at 28.632 dB, exceeding U-shape by 1.330 dB, and the highest SSIM at 0.895, compared with 0.882 for DNnet and 0.795 for FGDPA. The results show dataset-dependent strengths: DNnet leads both UIEB metrics, while PAUNet leads both metrics on EUVP and LSUI.

**Figure 6:**
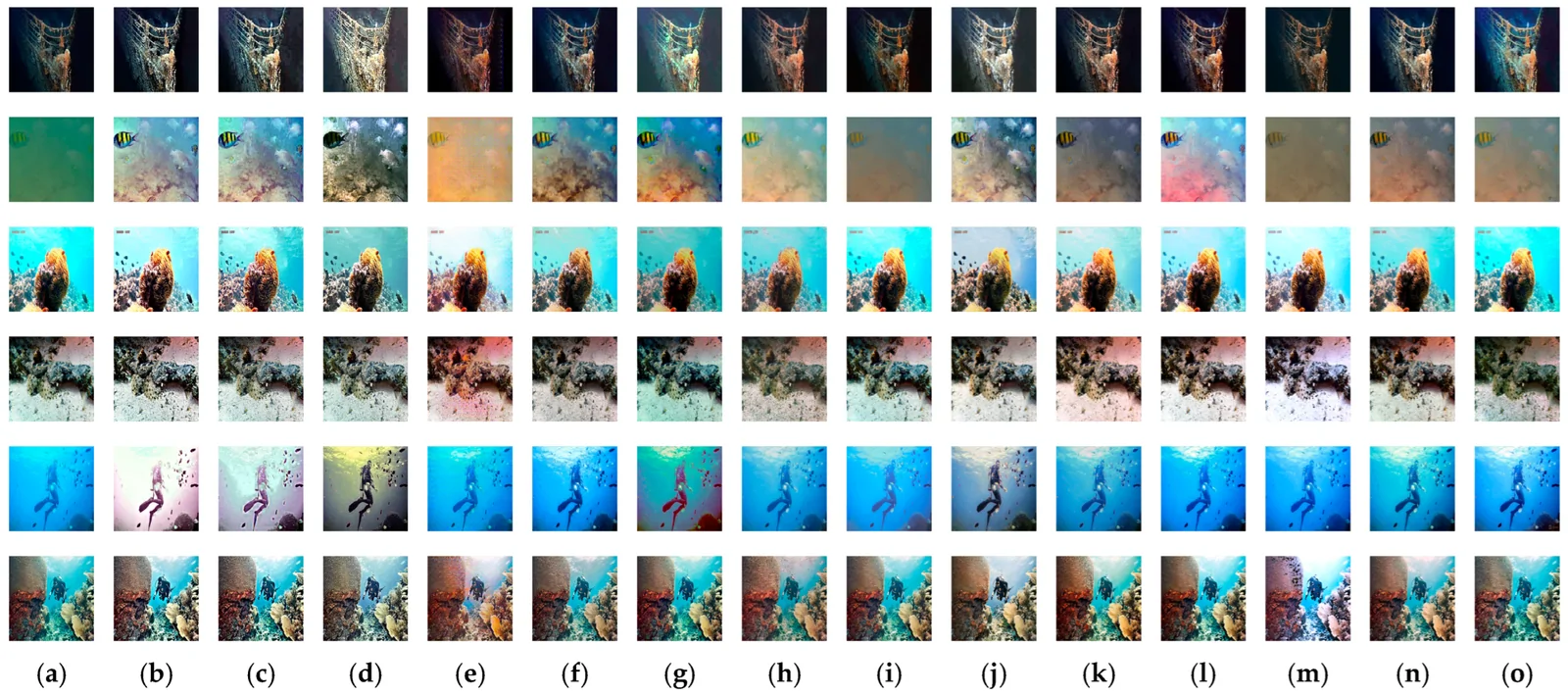
Visual comparison on UIEB [12]: (**a**) input; (**b**) MLLE; (**c**) WWPF; (**d**) WFAC; (**e**) FUnIE-GAN; (**f**) Water-Net; (**g**) USUIR; (**h**) U-shape; (**i**) CCL-Net; (**j**) TUDA; (**k**) LU2Net; (**l**) DNnet; (**m**) FGDPA; (**n**) PAUNet; and (**o**) reference.

**Figure 7:**
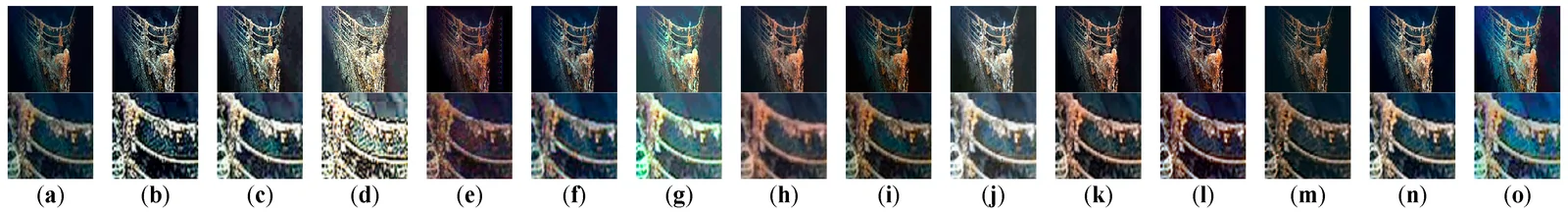
Full images and matched detail crops of the first UIEB [12] example in Figure 6. (**a**) input; (**b**) MLLE; (**c**) WWPF; (**d**) WFAC; (**e**) FUnIE-GAN; (**f**) Water-Net; (**g**) USUIR; (**h**) U-shape; (**i**) CCL-Net; (**j**) TUDA; (**k**) LU2Net; (**l**) DNnet; (**m**) FGDPA; (**n**) PAUNet; and (**o**) reference.

**Figure 8:**
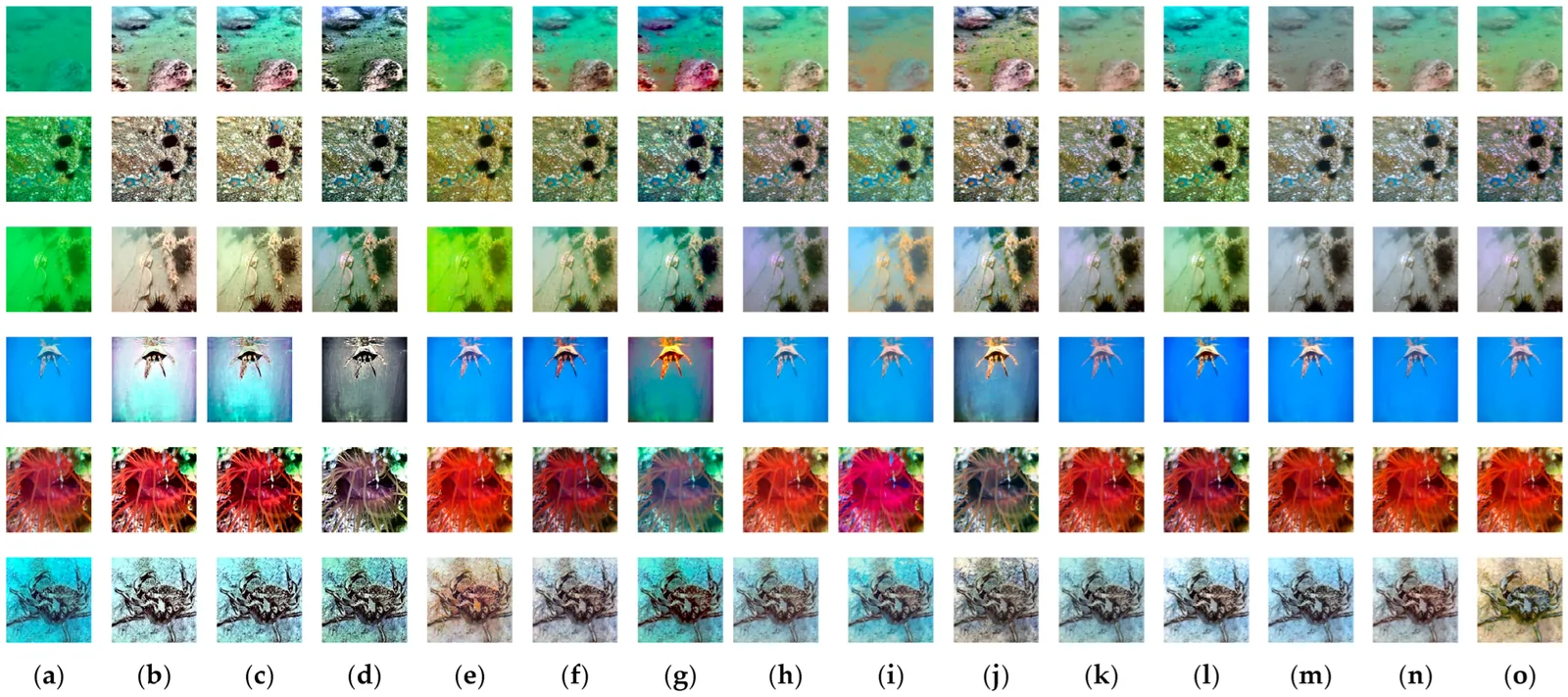
Visual comparison on EUVP [13]: (**a**) input; (**b**) MLLE; (**c**) WWPF; (**d**) WFAC; (**e**) FUnIE-GAN; (**f**) Water-Net; (**g**) USUIR; (**h**) U-shape; (**i**) CCL-Net; (**j**) TUDA; (**k**) LU2Net; (**l**) DNnet; (**m**) FGDPA; (**n**) PAUNet; and (**o**) reference.

**Figure 9:**
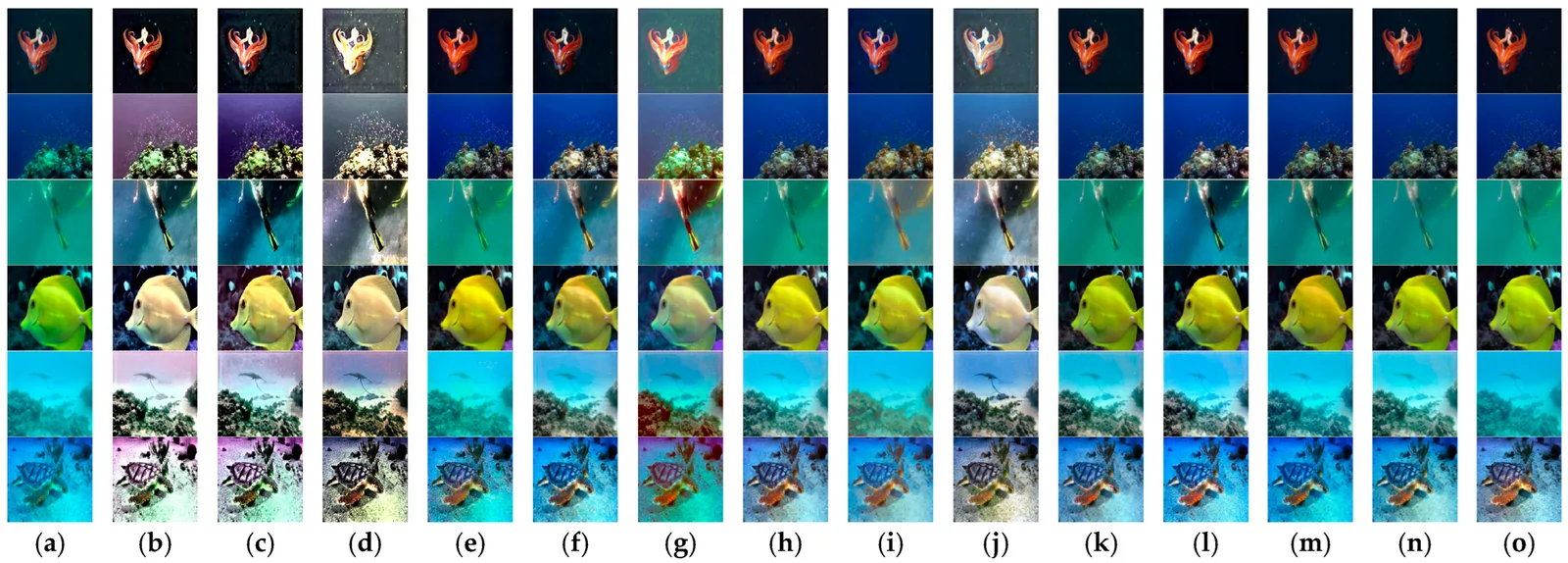
Visual comparison on LSUI [15]: (**a**) input; (**b**) MLLE; (**c**) WWPF; (**d**) WFAC; (**e**) FUnIE-GAN; (**f**) Water-Net; (**g**) USUIR; (**h**) U-shape; (**i**) CCL-Net; (**j**) TUDA; (**k**) LU2Net; (**l**) DNnet; (**m**) FGDPA; (**n**) PAUNet; and (**o**) reference.

**Figure 10:**
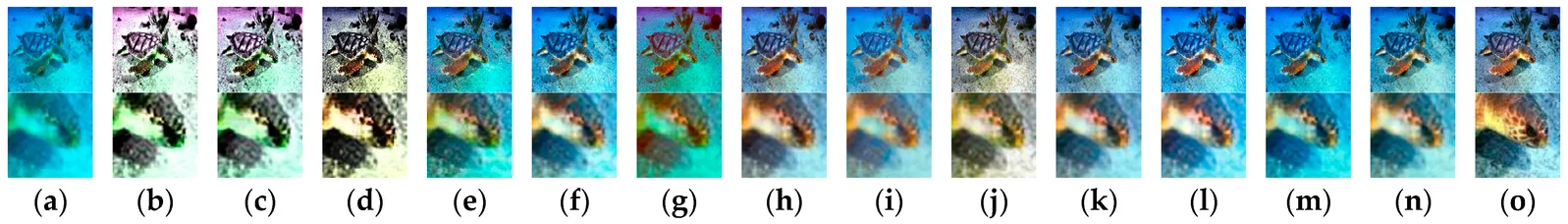
Full images and matched detail crops of the sixth LSUI [15] example in Figure 9. (**a**) input; (**b**) MLLE; (**c**) WWPF; (**d**) WFAC; (**e**) FUnIE-GAN; (**f**) Water-Net; (**g**) USUIR; (**h**) U-shape; (**i**) CCL-Net; (**j**) TUDA; (**k**) LU2Net; (**l**) DNnet; (**m**) FGDPA; (**n**) PAUNet; and (**o**) reference.

Figure 6 compares full-image enhancements on UIEB, with magnified details in Figure 7. Figure 8 and Figure 9 show full-image comparisons on EUVP and LSUI, respectively, with LSUI details in Figure 10. The displayed PAUNet outputs show reduced blue-green casts and improved visibility of scene structures. Some competing outputs retain color imbalance or produce stronger local contrast. These examples complement the numerical results by showing differences in color rendering and detail preservation.

Figure 7 provides a matched detail comparison of the rope-and-hull region in the first UIEB example shown in Figure 6. The enlarged views make the rope contours, attached surface texture, and local color differences easier to inspect. PAUNet reduces the blue-green shading and improves the visibility of the rope boundaries. The other outputs differ in highlight intensity, local contrast, and residual color cast. Every method uses the same relative crop coordinates and display size. No sharpening or color/contrast adjustment is applied to the displayed images.

Figure 10 examines the shell-to-head boundary of the turtle in the sixth LSUI example shown in Figure 9. The fourfold crop enlargement exposes differences in the dark shell markings, orange head region, and surrounding water. PAUNet retains the visible contour and color separation, although the small-scale texture remains soft at the available image resolution. These localized observations complement the quantitative comparisons in Table 1.

### 4.2. Evaluation Without Reference Images

Table 2 reports no-reference scores on the complete UIEB Challenging-60 set. Across all 13 methods, PAUNet ranks third in UIQM at 2.869, after FUnIE-GAN (3.007) and TUDA (2.917). Its UCIQE of 0.471 is tied with WFAC for fourth place at the displayed precision, behind WWPF (0.478), MLLE (0.477), and TUDA (0.472). DNnet obtains 2.548 UIQM and 0.398 UCIQE, while FGDPA obtains 2.726 UIQM and 0.390 UCIQE. PAUNet exceeds DNnet and FGDPA on both reported no-reference metrics. FUnIE-GAN and WWPF provide the highest overall UIQM and UCIQE, respectively.

**Figure 11:**
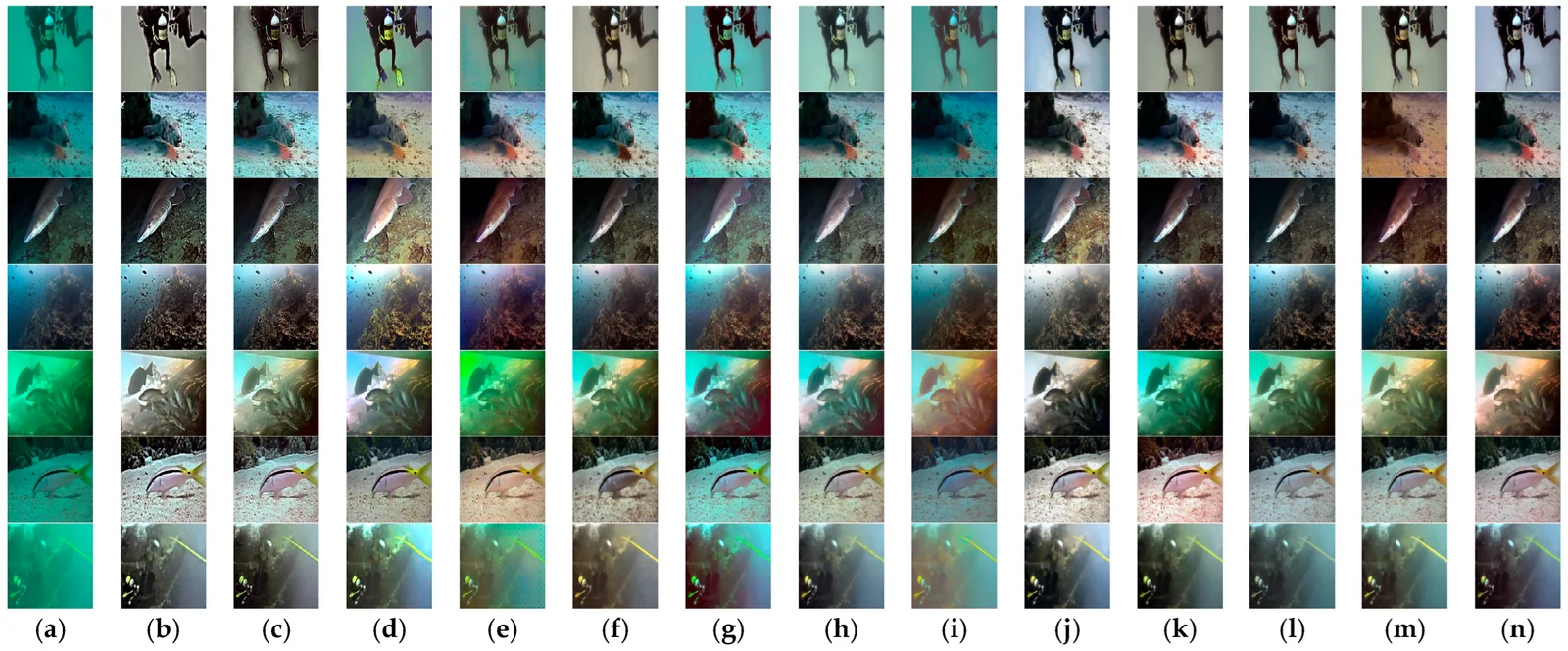
Visual comparison on UIEB Challenging-60 [12]: (**a**) input; (**b**) MLLE; (**c**) WWPF; (**d**) WFAC; (**e**) FUnIE-GAN; (**f**) Water-Net; (**g**) USUIR; (**h**) U-shape; (**i**) CCL-Net; (**j**) TUDA; (**k**) LU2Net; (**l**) DNnet; (**m**) FGDPA; and (**n**) PAUNet.

Representative challenging examples are shown in Figure 11. PAUNet reduces visible color casts and improves contrast without the same degree of saturation seen in some comparison outputs.

### 4.3. Ablation Studies

#### 4.3.1. Contributions of the Network Modules

In “w/o PADW”, encoder/decoder filtering uses all channels. In “w/o MASA”, a single k = 5 PADW branch with surrounding pointwise convolutions replaces the multiscale bottleneck. In “w/o CSAM”, skip features are concatenated without recalibration. The remaining structure is unchanged. Table 3 reports eight UIEB configurations: the full model; three single replacements; three paired replacements; and “w/o all”, which applies all three replacements. A plus sign joins the modules replaced together.

In Table 3, the full model achieves 21.508 dB PSNR, 0.751 SSIM, 3.587 UIQM, and 0.583 UCIQE, with the highest PSNR, UIQM, and UCIQE. Replacing PADW, MASA, or CSAM reduces PSNR by 2.342, 1.491, or 1.000 dB and SSIM by 0.021, 0.015 or 0.010, respectively. The “w/o PADW + MASA”, “w/o PADW + CSAM”, and “w/o MASA + CSAM” variants have PSNR values of 20.875, 20.513, and 21.225 dB, respectively, below the full model by 0.633, 0.995, and 0.283 dB. Their SSIM values are 0.784, 0.785, and 0.791, exceeding the full model by 0.033, 0.034, and 0.040. The “w/o all” configuration yields 20.712 dB and 0.732 SSIM, below the full model by 0.796 dB and 0.019. Thus, the full configuration leads three metrics, while SSIM favors some paired-removal variants.

**Figure 12:**
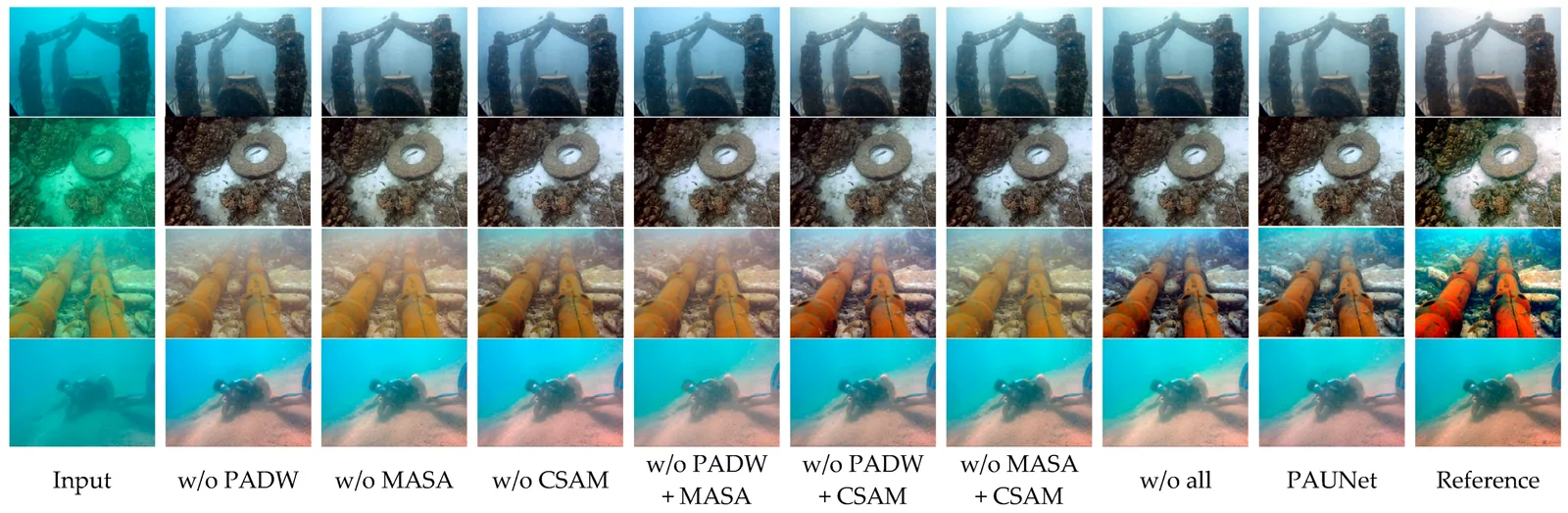
Module ablation on UIEB [12]. From left to right: Input, w/o PADW, w/o MASA, w/o CSAM, w/o PADW + MASA, w/o PADW + CSAM, w/o MASA + CSAM, w/o all, PAUNet, and Reference.

Figure 12 shows more complete color correction and clearer local structure for the full model in these scenes. The visual differences complement the quantitative module ablations.

#### 4.3.2. Partial-Channel Ratio

Table 4 evaluates r ∈ {1/8, 1/4, 1/2, 3/4, 1}. The symbol r denotes a fraction, whereas C_p_ denotes a channel count. Parameter count and reported FLOPs increase with r because more channels participate in axial filtering. The r = 1/2 configuration achieves the highest PSNR and SSIM, with 64,495 parameters and 0.493 GFLOPs. At r = 1, the computational cost rises to 0.512 GFLOPs and PSNR decreases to 19.166 dB. Relative to r = 1, using r = 1/2 reduces the whole-network computational cost by approximately 3.7%, calculated as (0.512 − 0.493)/0.512. This differs from the 50% reduction in the axial spatial operator because other network operations remain. The quality does not increase monotonically with r; r = 1/2 provides the best observed quality–cost balance among the tested configurations.

The smaller ratios further reduce computational cost but also decrease reconstruction accuracy: r = 1/8 reaches 17.725 dB and r = 1/4 reaches 19.374 dB. Increasing the ratio from 1/2 to 3/4 also reduces PSNR to 20.049 dB, despite adding computation. These comparisons motivate the selected partial ratio for the present architecture and training setting.

**Figure 13:**
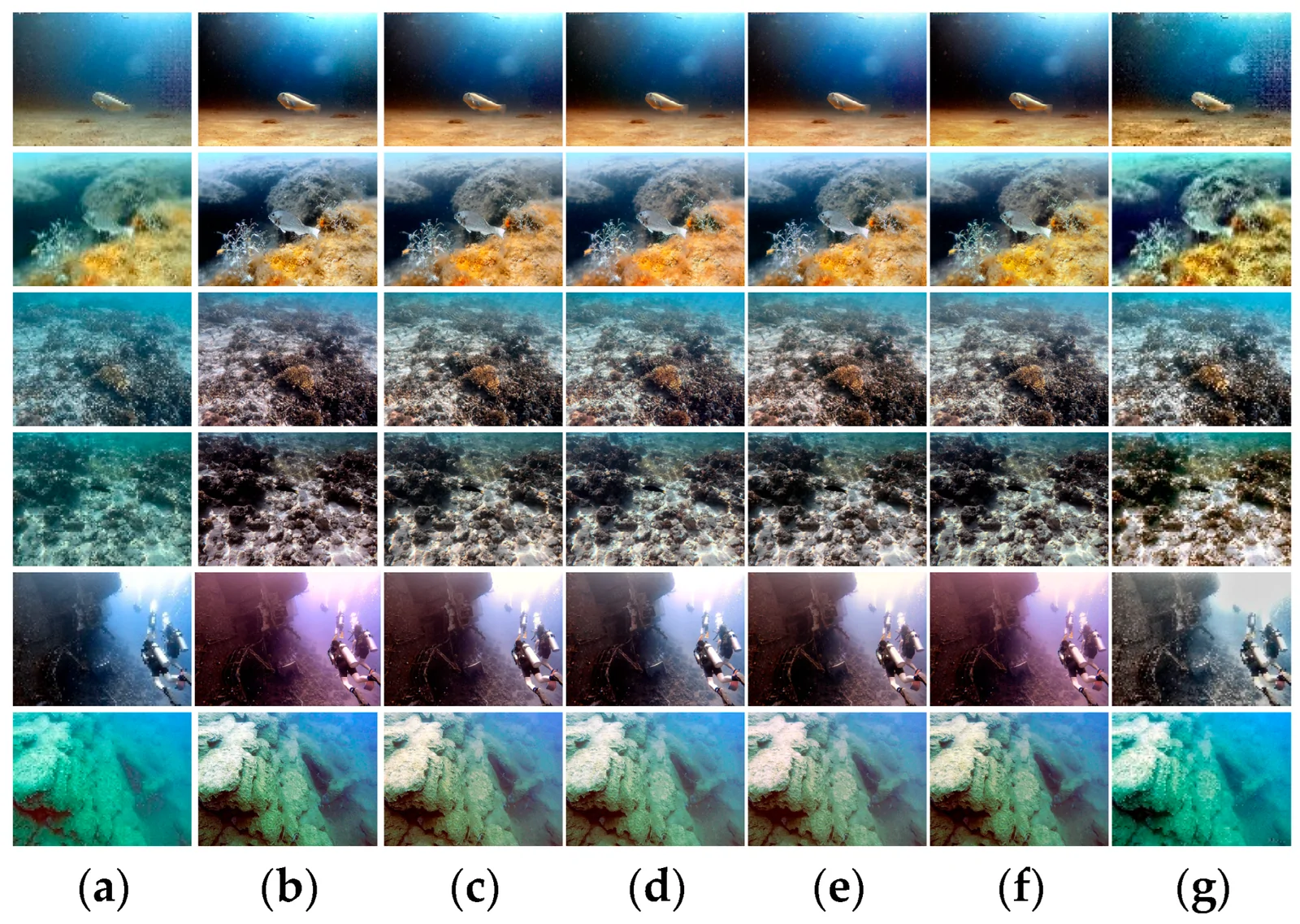
Effect of the PADW partial ratio on UIEB [12]: (**a**) input; (**b**) r = 1/8; (**c**) r = 1/4; (**d**) r = 1/2; (**e**) r = 3/4; (**f**) r = 1; and (**g**) reference.

The visual comparison in Figure 13 shows the corresponding differences in color recovery and edge detail.

#### 4.3.3. Loss Components

Loss ablations are evaluated on the LSUI test split by removing one loss term at a time and retaining the remaining weights. Table 5 includes removal of the Charbonnier, Lab, LCH, SSIM, and VGG terms. The full objective achieves 28.632 dB PSNR, 0.895 SSIM, 1.250 UIQM, and 0.634 UCIQE, the highest values among these variants.

**Figure 14:**
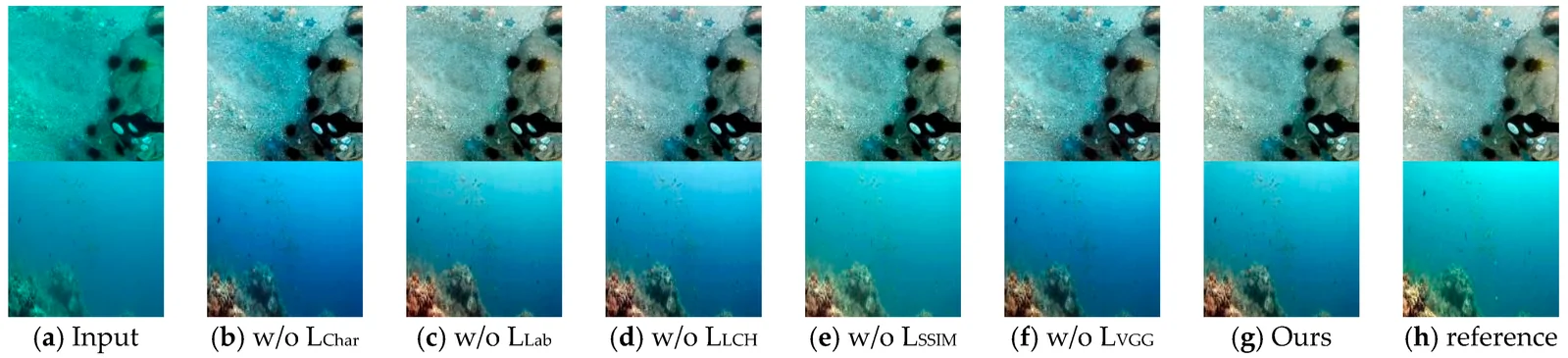
Loss ablation on LSUI [15]: (**a**) input; (**b**) w/o L_Char_; (**c**) w/o L_Lab_; (**d**) w/o L_LCH_; (**e**) w/o L_SSIM_; (**f**) w/o L_VGG_; (**g**) Ours (full loss); and (**h**) reference.

Removing VGG supervision produces the largest PSNR decrease, 4.551 dB. Removing the color terms also reduces reconstruction accuracy: the decreases are 2.594 dB for Lab and 3.305 dB for LCH. The full-loss examples in Figure 14 exhibit balanced color and a clearer structure. Under the fixed coefficients in Equation (19), each loss-removal variant has lower PSNR and SSIM than the full objective, supporting the contribution of each term within this weighted configuration.

### 4.4. Model Complexity and Inference Speed

Table 6 compares model complexity and inference speed on an NVIDIA RTX 3090. DNnet has 34,742 parameters and achieves 227.55 FPS, with a latency of 4.39 ms. FGDPA has 4234 parameters and achieves 559.39 FPS, with a latency of 1.79 ms, achieving the fewest parameters and highest throughput among the compared methods. PAUNet has 64,495 parameters and a reported computational cost of 0.493 GFLOPs, achieving 288.95 FPS, with a latency of 3.46 ms.

Relative to USUIR, PAUNet uses approximately 71% fewer parameters and 98% fewer FLOPs. Its latency is 3.46 ms, compared with 3.39 ms for USUIR; thus, a lower operation count does not correspond to lower measured latency. PAUNet, nevertheless, offers substantially higher PSNR on the three paired evaluations. Compared with LU2Net, PAUNet reduces parameters by approximately 63% and FLOPs by 83%, while increasing measured throughput from 183.69 to 288.95 frames per second. The results show an efficient quality–size trade-off, with latency assessed directly on the stated hardware.

### 4.5. Real-World Underwater Imaging Test

**Figure 15:**
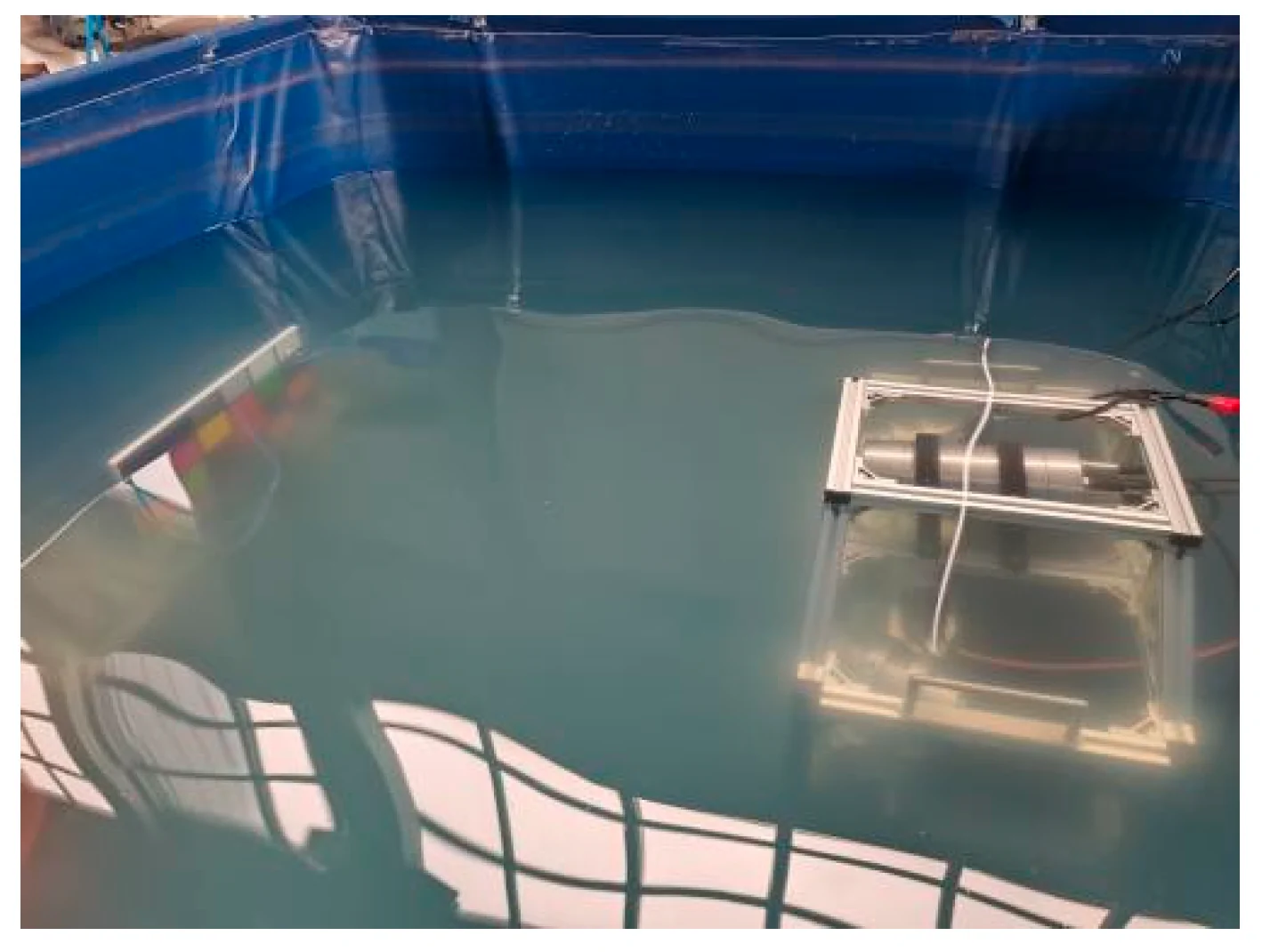
Self-developed underwater imaging platform with a sealed camera housing, auxiliary lighting, and a support frame.

The custom-built pool-test imaging system combines a sealed cylindrical camera housing, auxiliary lighting, and a rigid support frame (Figure 15). The trained network enhances individual 256 × 256 video frames without multiframe buffering or temporal post-processing.

On the NVIDIA Jetson Xavier NX (NVIDIA Corporation, Santa Clara, CA, USA), PAUNet achieves a measured network-inference throughput of 74.55 FPS in 15 W six-core mode, using GPU inference with TensorRT FP16, a batch size of 1, and 256 × 256 inputs. Measurement uses 200 warm-up forwards followed by three repetitions of 1000 timed forwards. The reported throughput excludes image preprocessing and post-processing.

**Figure 16:**
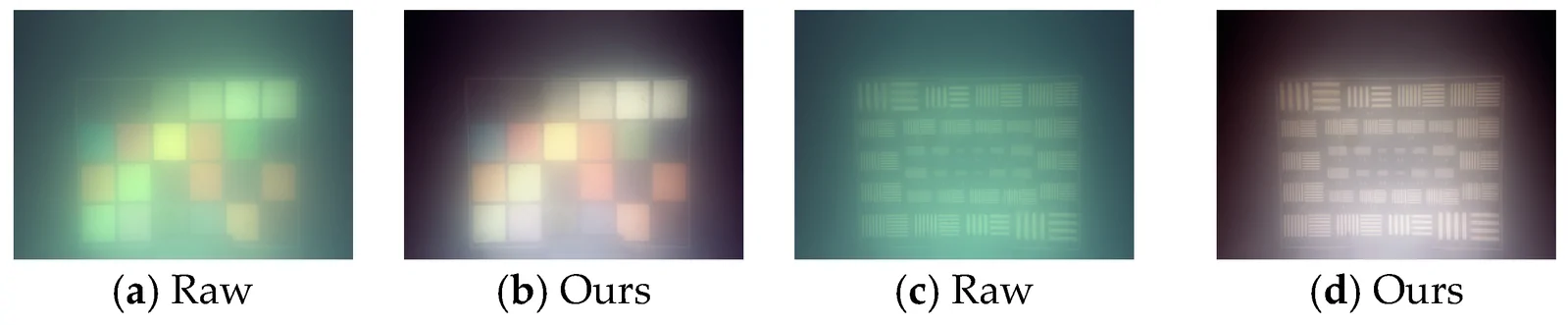
Raw and PAUNet-enhanced pool images: (**a**,**b**) color-chart images before and after enhancement; and (**c**,**d**) resolution-chart images before and after enhancement.

Figure 16 presents raw and enhanced frames containing a color chart and a resolution chart. The examples illustrate changes in color appearance and the visibility of local detail. Because matched reference images are unavailable, these images provide a qualitative assessment.

## 5. Discussion

The experiments indicate that a small enhancement model can retain useful reconstruction capacity when spatial computation, multiscale processing, and skip-feature fusion are allocated selectively. PADW reduces the cost of each axial spatial operator, while the pointwise projections preserve channel interaction. MASA operates at the lowest resolution and adds scale diversity without repeating the same multibranch structure throughout the network. The module ablation favors the full configuration in PSNR, UIQM, and UCIQE, although three paired-removal variants obtain higher SSIM. The full loss leads all four metrics in Table 5.

Across all methods in Table 1, PAUNet has the highest PSNR and SSIM on both EUVP and LSUI. DNnet leads both UIEB metrics, while PAUNet ranks second in UIEB PSNR. On Challenging-60, PAUNet ranks third in UIQM and tied fourth in UCIQE across the complete comparison set. These results indicate that performance depends on the dataset and metric. The PAUNet/USUIR comparison in Table 6 also shows that fewer parameters and FLOPs do not necessarily give lower GPU latency. Hardware conclusions are limited to the methods and conditions actually measured.

PAUNet uses channel compression in CA and the internal MASA projections and contains 64,495 parameters. Its full-image and enlarged comparisons show changes in color rendering and local structure, while repeated-run statistics and capacity-matched training controls require additional results. Future work will examine temporal consistency and a wider range of underwater imaging conditions.

## 6. Conclusions

This study presented PAUNet, a lightweight underwater image enhancement network combining partial axial depthwise convolution, multiscale bottleneck attention, and recalibrated skip connections. A joint objective constrains reconstruction, structure, perceptual features, and color. Across all methods in Table 1, PAUNet achieves the highest PSNR on EUVP and LSUI, at 28.696 and 28.632 dB, respectively. Its UIEB PSNR of 21.508 dB ranks second to DNnet. PAUNet achieves the highest SSIM on EUVP and LSUI, at 0.843 and 0.895, respectively. On Challenging-60, it ranks third in UIQM and tied fourth in UCIQE. PAUNet contains 64,495 parameters, requires a reported 0.493 GFLOPs and reaches 288.95 frames per second on an NVIDIA RTX 3090. Its measured network-inference throughput on an NVIDIA Jetson Xavier NX is 74.55 FPS, with TensorRT FP16 at 256 × 256 resolution and batch size one. The comparisons, ablations, enlarged detail views, and pool-scene results support PAUNet as a compact component for underwater visual enhancement, with quality advantages that vary across datasets and metrics.

## Figures and Tables

**Table 1:** Full-reference comparison on the same fixed UIEB and EUVP subsets (100 image pairs each) and the LSUI test split. PSNR is in dB. Bold indicates the highest value among all listed methods.

Method	UIEB PSNR	UIEB SSIM	EUVP PSNR	EUVP SSIM	LSUI PSNR	LSUI SSIM
MLLE	17.508	0.738	16.622	0.625	17.558	0.680
WWPF	18.532	0.757	17.267	0.612	18.275	0.689
WFAC	19.975	0.775	15.349	0.528	16.997	0.567
FUnIE-GAN	17.247	0.584	28.506	0.810	25.193	0.729
Water-Net	20.739	0.767	23.796	0.772	24.258	0.830
USUIR	20.371	0.818	19.735	0.661	20.907	0.726
U-shape	18.498	0.691	27.181	0.825	27.302	0.845
CCL-Net	20.454	0.719	27.579	0.736	24.754	0.788
TUDA	20.511	0.813	19.627	0.681	19.757	0.706
LU2Net	19.531	0.739	26.445	0.793	25.475	0.793
DNnet	**22.454**	**0.891**	18.879	0.836	22.928	0.882
FGDPA	20.071	0.859	18.734	0.779	24.974	0.795
PAUNet	21.508	0.751	**28.696**	**0.843**	**28.632**	**0.895**

**Table 2:** No-reference comparison on the same 60 UIEB Challenging-60 images for all listed methods. Bold indicates the highest value in each metric across the complete comparison set.

Method	UIQM	UCIQE
MLLE	2.485	0.477
WWPF	2.617	**0.478**
WFAC	2.738	0.471
FUnIE-GAN	**3.007**	0.411
Water-Net	2.681	0.445
USUIR	2.716	0.462
U-shape	2.783	0.423
CCL-Net	2.350	0.384
TUDA	2.917	0.472
LU2Net	2.578	0.438
DNnet	2.548	0.398
FGDPA	2.726	0.390
PAUNet	2.869	0.471

**Table 3:** Module ablation: all four metrics use the same 100 paired UIEB images; Table 2 separately evaluates Challenging-60. “w/o” denotes the stated replacement. Bold indicates the best value.

Variant	PSNR (dB)	SSIM	UIQM	UCIQE
w/o PADW	19.166	0.730	3.124	0.556
w/o MASA	20.017	0.736	3.285	0.561
w/o CSAM	20.508	0.741	3.401	0.571
w/o PADW + MASA	20.875	0.784	3.467	0.562
w/o PADW + CSAM	20.513	0.785	3.459	0.567
w/o MASA + CSAM	21.225	**0.** **7** **91**	3.508	0.563
w/o all	20.712	0.732	3.408	0.547
PAUNet	**21.508**	0.751	**3.587**	**0.583**

**Table 4:** Partial-ratio ablation evaluated on UIEB. Bold indicates the highest PSNR and SSIM.

Ratio r	Params (K)	FLOPs (G)	PSNR (dB)	SSIM
1/8	63.487	0.479	17.725	0.725
1/4	63.823	0.484	19.374	0.740
1/2	64.495	0.493	**21.508**	**0.751**
3/4	65.167	0.503	20.049	0.747
1	65.839	0.512	19.166	0.730

**Table 5:** Loss ablation on the LSUI test split. Bold indicates the best value.

Objective	PSNR (dB)	SSIM	UIQM	UCIQE
w/o L_Char_	26.214	0.874	0.823	0.591
w/o L_Lab_	26.038	0.873	1.037	0.619
w/o L_LCH_	25.327	0.879	1.081	0.613
w/o L_SSIM_	25.143	0.876	0.817	0.597
w/o L_VGG_	24.081	0.878	0.886	0.607
Full loss	**28.632**	**0.895**	**1.250**	**0.634**

**Table 6:** Model complexity and inference speed on an NVIDIA RTX 3090. Bold indicates the best value.

Method	Params (M)	FLOPs (G)	Time (ms)	FPS
FUnIE-GAN	7.020	10.239	4.75	210.39
Water-Net	1.091	142.845	5.55	180.22
USUIR	0.226	29.494	3.39	295.03
U-shape	22.817	2.983	31.27	31.98
CCL-Net	0.451	214.339	12.78	78.22
TUDA	1.975	85.433	14.53	68.83
LU2Net	0.176	2.859	5.44	183.69
DNnet	0.035	0.511	4.39	227.55
FGDPA	**0.004**	0.503	**1.79**	**559.39**
PAUNet	0.064	**0.493**	3.46	288.95

## Data Availability

The datasets used in this study are publicly available from the sources cited for UIEB [12], EUVP [13], and LSUI [15]. Data-partition and sampling seeds, fixed evaluation subsets, and metric settings are specified in Section 3.5 and Section 3.6.
